# Are AAMA and GAMA Levels in Urine after Childbirth a Suitable Marker to Assess Exposure to Acrylamide from Passive Smoking during Pregnancy?—A Pilot Study

**DOI:** 10.3390/ijerph17207391

**Published:** 2020-10-11

**Authors:** Hanna Mojska, Iwona Gielecińska, Edyta Jasińska-Melon, Joanna Winiarek, Włodzimierz Sawicki

**Affiliations:** 1 Department of Nutrition and the Nutritional Value of Food, National Institute of Public Health-National Instutute of Hygiene, Chocimska 24, 00-791 Warsaw, Poland; ejasinska@pzh.gov.pl; 2 Department of Food Safety National Institute of Public Health—National Institute of Hygiene, Chocimska 24, 00-791 Warsaw, Poland; igielecinska@pzh.gov.pl; 3 Chair and Department of Obstetrics, Gynecology and Gynecological Oncology of Medical University of Warsaw, Kondratowicza 8, 03-242 Warsaw, Poland; joanna.winiarek@wum.edu.pl (J.W.); saw55@wp.pl (W.S.)

**Keywords:** acrylamide, AAMA and GAMA, passive smoking, pregnancy, birth parameters

## Abstract

Introduction: Acrylamide (AA) is a “probably carcinogenic to humans” monomer that can form in heated starchy food and in tobacco smoke. N-Acetyl-S-(2-carbamoylethyl)-L-cysteine (AAMA) and N-Acetyl-S-(2-carbamoyl-2-hydroxyethyl)-L-cysteine (GAMA), acrylamide metabolites in urine, are recognized as good markers of exposure to acrylamide. Aim: The aim of the study is a preliminary assessment whether the levels of AAMA and GAMA in urine after childbirth are good markers of acrylamide exposure due to passive smoking during pregnancy. Material and method: The study group consisted 67 non-smokers and 10 passive-smoker women during pregnancy. AAMA and GAMA levels in urine samples were determined using liquid chromatography coupled with tandem mass spectrometry (LC-MS/MS). Results: The median AAMA levels in urine of non-smoking and passively smoking women were 30.7 μg/g creatinine and 25.2 μg/g creatinine, respectively. Much lower values were determined for GAMA: 11.4 μg/g creatinine and 10.3 μg/g creatinine, respectively. There is no significant difference between AAMA and GAMA content in urine samples between both groups of women as well as in the anthropometric parameters of newborns between those two groups of mothers. Conclusion: Our pilot study did not confirm that postpartum AAMA and GAMA concentrations in urine are good markers of exposure to acrylamide from passive smoking during pregnancy. It is probably due to the different ways of acrylamide absorption from tobacco smoke by active and passive smokers. Exposure of pregnant women to acrylamide from passive smoking requires further research.

## 1. Introduction

Smoking is one of the greatest health risks, and its negative effects are observed both among active and passive smokers, also in early life [1]. Although there has been a reduction in smoking among pregnant women in recent years, an average of more than 10% of pregnant women still smoke in Europe, ranging from less than 5% (Lithuania, Sweden, Norway) to more than 18% (Spain) [2]. 

Smoking during pregnancy can lead, among other things, to a reduction in the birth weight and length of the child, premature birth, degenerative changes in the placenta and consequently an increased risk of abnormal newborn development [3]. It has also been confirmed that smoking 10 or more cigarettes a day by a pregnant woman significantly (OR = 1.79, 95% CI = 1.39–2.31) increases the risk of sight defect (strabismus) in the offspring, manifested by weakened eye muscles [4]. A number of studies also highlight the impact of smoking during pregnancy on the increase in the risks of premature deaths and deaths due to Sudden Infant Death Syndrome (SIDS) [5]. The results of a recently published meta-analysis covering 22 studies (111,712 newborns in total) indicate a correlation between smoking during pregnancy and an increase in the risk of cryptorchidism in male infants by 1.18 times (OR 1.18, 95%, CI 1.12–1.24, *p* < 0.00001) compared to non-smoking mothers [6]. Passive smoking can have as negative health effects as active smoking [3,7]. It is estimated that exposure to environmental tobacco smoke is equivalent to smoking 8 cigarettes per day. In addition, it appears that a passive smoker inhales more of the toxic substances contained in the lateral stream of tobacco smoke than a smoker exposed to the main stream of smoke [8]. Tobacco smoke contains more than 5000 of chemical compounds, including over 40 known carcinogens and a number of toxic substances [9]. Among the toxic compounds present in tobacco smoke there is also acrylamide [10,11]. Cigarette smoking appears to be the second most important source of exposure, following food, to this compound [12,13,14,15]. 

Acrylamide in food is formed by the Maillard reaction between free asparagine and reducing sugars, mainly glucose and fructose, at temperatures above 120 °C [16]. The main source of acrylamide in the diet are potato products such as fries and chips, cereal products such as bread, breakfast cereals, cookies and coffee and its substitutes. In Europe, estimated exposure to acrylamide from food ranges from 0.4 to 1.9 µg/kg b.w./day [17]. 

Numerous studies have shown that acrylamide is a neurotoxic compound and can cause damage to the central and peripheral nervous system, both in experimental animals and in humans exposed to the compound at work [12,18]. In animal studies, an increase in the incidence of tumors of many organs, including testes, heart, uterus, adrenals, thyroid, mammary gland, oral cavity and skin after acrylamide is applied in drinking water has been observed [19,20]. The International Agency for Research on Cancer [21] already in 1994 classified acrylamide as “probably carcinogenic to humans” (Group 2A), concluding that although there is limited evidence of the carcinogenic effects of acrylamide in humans, the evidence of such effects in experimental animal studies is well documented. 

Acrylamide is rapidly absorbed and owing to its very good solubility in water, rapidly distributed to various tissues. It may penetrate the placental barrier [22] and pose a risk to the developing foetus. So far, three epidemiological studies have shown a connection between the uptake of acrylamide from food during pregnancy and the low birth weight and length of their children as well as the increased risk of small for gestational age (SGA) [23,24,25]. Acrylamide is metabolised through two main metabolic pathways: epoxidation to glycidamide and glutathione conjugation to mercapturic acids. The conversion of acrylamide to glycidamide, its main metabolite, is catalysed by an enzyme of cytochrome P450 (isoenzyme CYP2E1) [26]. Both acrylamide and glycidamide form adducts with haemoglobin [13,27]. Both compounds are conjugated to glutathione using S-transferase glutathione enzymes (GST): acrylamide to N-Acetyl-S-(2-carbamoylethyl)-L-cysteine (AAMA) and glycidamide to N-Acetyl-S-(2-carbamoyl-2-hydroxyethyl)-L-cysteine (GAMA) and N-acetyl-S-(3-amino-2-hydroxy-3-oxopropyl)-cysteine. Metabolites of acrylamide and glycidamide in the form of mercapturic acid derivatives are excreted in urine [28]. So far, only the United States Environmental Protective Agency (USEPA) has developed an official position on the relationship between AAMA and GAMA levels in urine and cancer risk. The USEPA reference doses for cancer, based on an additional lifetime cancer risk of 1 × 10^−4^ and 1 × 10^−6^, correspond to 2 µg AAMA/g creatinine and 0.02 µg AAMA/g creatinine, respectively [29].

Until now, several studies [13,14,30,31,32] have confirmed a positive relationship between active smoking and AAMA and GAMA levels in urine of the examined persons. However, the results of studies in passive smokers are inconclusive [32,33,34,35]. A significant (*p* = 0.028) positive correlation between AAMA and urinary cotinine (a recognized marker of exposure to tobacco smoke) urine has also been confirmed only in active smokers [36].

In our previous study [37], we found significantly (*p* < 0.01) higher levels of AAMA and GAMA in the urine of postpartum women who smoked while pregnant compared to pregnant non-smokers. The aim of our current study is a preliminary assessment whether the levels of AAMA and GAMA in urine after childbirth are good markers of acrylamide exposure due to second-hand smoking during pregnancy. In addition, we wanted to assess whether there is a link between exposure to acrylamide resulting from passive smoking by pregnant women and anthropometric parameters of newborns. 

## 2. Material 

### 2.1. The Study Group

Women recruitment, inclusion and exclusion criteria were essentially the same as described before [37]. In brief, the study group was recruited among healthy women who gave birth in the obstetric ward in the Chair and Department of Obstetrics, Gynecology and Gynaecological Oncology in Medical University in Warsaw, during three consecutive months. The criterion for inclusion in the research group was the health of the mother and a written consent to participate in the study. The criterion of exclusion were chronic diseases in the mother and genetic defects in the child. All women were asked to complete a sociodemographic survey that include, among others, questions about age, pregnancy length and smoking habits during pregnancy. From the overall group of 93 women, for the purposes of the current study, 5 women who declared that they actively smoked throughout their pregnancy were excluded. We also excluded 11 women from this group who smoked actively in the first period of pregnancy until they found out that they were pregnant. From the remaining group of 77 women, based on a sociodemographic interview conducted after childbirth, a group of 10 women (13%) were selected who declared that they were passively exposed to tobacco smoke throughout their pregnancy. The number of cigarettes smoked in the presence of the abovementioned pregnant women was on average 7.2 cigarettes/day, ranging from 0.2 to 20 cigarettes/day. The lowest number of cigarettes (0.2 cigarette/day) was calculated on the basis of information given by one of the women that on average 5 cigarettes/month were smoked in her presence. 

Women who participated in our study stayed in the hospital for 2–5 days depending on their health status and/or health status of their child. In all women, measurements of actual weight were performed. Data on birth weight and length of children of the participating women and information on the Apgar score were also collected.

All of the procedures performed in this study were in accordance with the ethical standard from the 1964 Helsinki declaration and its later amendments. Ethical approval was obtained from the Ethics Committee at the National Food and Nutrition Institute in Warsaw, Poland, and National Science Center (Project No. N N404 067740). Written consent was obtained from all participants. The characteristics of pregnant women passively smoking (*n* = 10) are shown in Table 1 and compared with the data for the remaining 67 women who declared that they have never smoked and were not exposed to tobacco smoke during pregnancy.

### 2.2. Urine Sampling

On day 2 to 5 after birth, depending on the length of hospital stay, each of the participants collected the morning urine sample (the first passed urine after the night) into a polypropylene container. Urine samples were immediately transferred to the laboratory of the Food and Nutrition Institute, Warsaw where they were frozen and stored at ~−70 °C until analysis.

### 2.3. Dietary Intake Assessment

On the same day on which morning urine sample was collected, a face-to-face interview was held about food consumption within the past 24 h. The portion size was verified with the use of an “Album of Photographs of Food Products and Dishes” [38].

## 3. Methods

### 3.1. Determination of AAMA and GAMA Levels in Urine 

The levels of AAMA and GAMA, acrylamide and glycidamide mercapturates in urine were determined by liquid chromatography coupled with tandem mass spectrometry (LC-MS/MS) as described earlier [37]. In short, after the urine samples had been thawed at room temperature and mixed, 4 mL was taken from each sample into a centrifuge tube, and 30 μL of a mixture of deuterated internal standards (d_4_-AAMA and d_3_-GAMA) was added. The samples were shaken mechanically, centrifuged and then cleaned on SPE columns. The supernatant was applied to the columns, which were previously conditioned with methanol, water and diluted formic acid. The tested compounds were eluted with 1% formic acid solution in HPLC methanol. The collected eluent, after evaporation to dryness, was dissolved in 0.5 mL of 0.1% formic acid solution LC-MS (v/v) and analysed with LC-MS/MS. 

Chromatographic separation was performed on the Phenomenex Kinetex 2.6u XB-C18 column using the Dionex UltiMate 3000 liquid chromatograph and the ABSciex 3200 QTrap mass spectrometer was used to determine AAMA and GAMA. The analysis was carried out using MRM technique (multiple reaction monitoring) in negative polarization. Ions of the tested compounds were monitored: m/z 233 → 104 (AAMA, CE: −22), m/z 236.9 → 108 (d_4_-AAMA, CE: −22), m/z 248.9 → 120.1 (GAMA, CE: −24) and m/z 252 → 119.9 (d_3_-GAMA, CE: −24) for the quantitative determination of the AAMA and GAMA content of urine, and m/z 233 → 58 (AAMA, CE: −54) and m/z 248.9 127.9 (GAMA, CE: −18) to verify the results obtained. Identification of metabolites was carried out on the basis of retention times and mass spectrum. The average of two parallel markings was taken as the result.

### 3.2. Determination of Urinary Creatinine Levels 

Urinary creatinine levels were measured by the VITROS CREA Slide method, which is performed using the VITROS CREA Slides and the VITROS Chemistry Products Calibrator Kit 1 on the VITROS-350 biochemistry analyser supplied by Ortho-Clinical Diagnostics (USA).

### 3.3. Estimation of Exposure to Acrylamide from Food Based on Food Consumption Data 

The dietary intake of acrylamide was calculated by taking into account our own earlier data on acrylamide content in food products in Poland [37]. Acrylamide content in each product consumed by the study subjects (in µg/kg) was multiplied by the size of portion of that product (in grams) estimated on the basis of the 24-h dietary recall, and the sum of the daily intake of acrylamide from all consumed products was then divided by the body weight individually for each woman participating in the study (in kilograms).

### 3.4. Estimation of Exposure to Acrylamide from Tobacco Smoke in a Group of Passive Smokers During Pregnancy

To estimate pregnant women’s exposure to acrylamide from cigarettes smoked in their presence, we used the results of our own research on the content of this compound in tobacco smoke condensate from the five most frequently purchased cigarette brands in Poland. The average acrylamide content was 679 ng/cigarette (range: 455–798 ng/cigarette) [15]. We used the average content of acrylamide in cigarettes for estimation purposes. 

Exposure to acrylamide from tobacco smoke for each woman who was the second-hand smoker during pregnancy was calculated by multiplying the number of cigarettes smoked in her presence by the average acrylamide content in the cigarette (679 ng/cigarette) and dividing by the body weight individually for each woman participating in the study (in kilograms).

### 3.5. Estimation of Exposure to Acrylamide from Food and Tobacco Smoke Based on AAMA and GAMA Levels in Urine of Women Post-Partum

The uptake of acrylamide (N_AA_) [µg/kg/b.w./day] based on the determined content of AAMA and GAMA metabolites in urine was calculated according to the formula [33]:

N_AA_ = [(a sum of AAMA + GAMA) × KW × M_AA_]/[0,5 × M_AAMA + GAMA_] (1)

where

Sum of AAMA + GAMA [µg/g creatinine];
KW—excretion of creatinine [mg/kg b.w./day];
M_AA_—molar mass of acrylamide (M_AA_ = 71);
0.5—the conversion factor [28];
M_AAMA + GAMA_—sum of molar masses AAMA and GAMA (M_AAMA + GAMA_ = 476).

For the calculation of creatinine excretion in urine, the actual analysed creatinine content in urine of each woman participating in the study, her body weight and the average amount of urine excreted daily by an adult woman were taken into account (1.4 L/day—assumed according to the Geigy Table, 1977) [39].

### 3.6. Statistical Analysis

The results of urinary levels of AAMA and GAMA expressed as a median and range (min– max) are presented in µg/l urine and in µg/g creatinine. The dietary intake of acrylamide and exposure to acrylamide via tobacco smoke are presented in µg/person/day and µg/kg b.w./day. The Mann–Whitney U-test was used for the comparison between passive smokers and non-smokers. Student’s t-test was used to compare anthropometric parameters of newborns. For the assessment of qualitative variables (“Type of labour” and “Labour order”) we used the χ2 test. Spearman’s correlation analysis was performed to determine the bivariate correlation between the exposure calculated on the basis of the level of AAMA and GAMA in the urine and acrylamide intake from the diet estimated on the basis of the 24-h dietary recall and via tobacco smoke in passive smokers. *p*-value = 0.05 was considered significant for the significance of differences or dependent correlation. Statistical analyses were carried out with the use of Statistica ver. 6.0. (StatSoft Inc., Tulsa, OK, USA).

## 4. Results

The characteristics of the women participating in the study are presented in Table 1. There were no significant differences in anthropometric and physiological parameters except for age. The average age of passive smokers was significantly lower (*p* < 0.05) compared to non-smokers. No significant differences in the average dietary intake of acrylamide were also found between the tested groups of women. 

Taking into account the average content of acrylamide in cigarettes in Poland, determined analytically in our earlier study [15], exposure to acrylamide from tobacco smoke in the group of passive smokers during pregnancy was calculated (Table 2). A significantly (*p* < 0.05) fivefold higher (µg/kg b.w./day) exposure was found in women who declared that during pregnancy 10, and more cigarettes were smoked daily in their presence compared to those in whose presence less than 10 pieces were smoked. 

**Figure 1:**
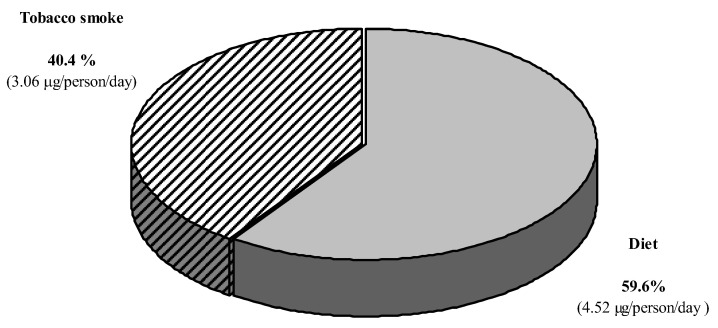
Percentage of acrylamide from different sources in women exposed to passive smoking. (*n* = 10).

As shown in Figure 1, the proportion of acrylamide absorbed from tobacco smoke represented on average nearly 60% of the total amount of this compound downloaded from food and from tobacco smoke in the group of passive smokers. 

The determined level of single acrylamide metabolites and their sum in urine of pregnant passive smokers after childbirth did not differ significantly from the level determined in urine of non-smoking women (Table 3). Furthermore, no significant differences in AAMA and GAMA levels in urine were found between passive smokers exposed to smoke from 10 and more cigarettes smoked daily in their presence and those where less than 10 were smoked daily in their presence. 

Comparing the exposure to acrylamide (µg/kg b.w./day) of non-smoking women and passive smokers, calculated on the basis 24-h dietary recall and acrylamide content in tobacco smoke and on the level of metabolites in urine, we found no significant differences between the study groups (Table 4). 

**Figure 2:**
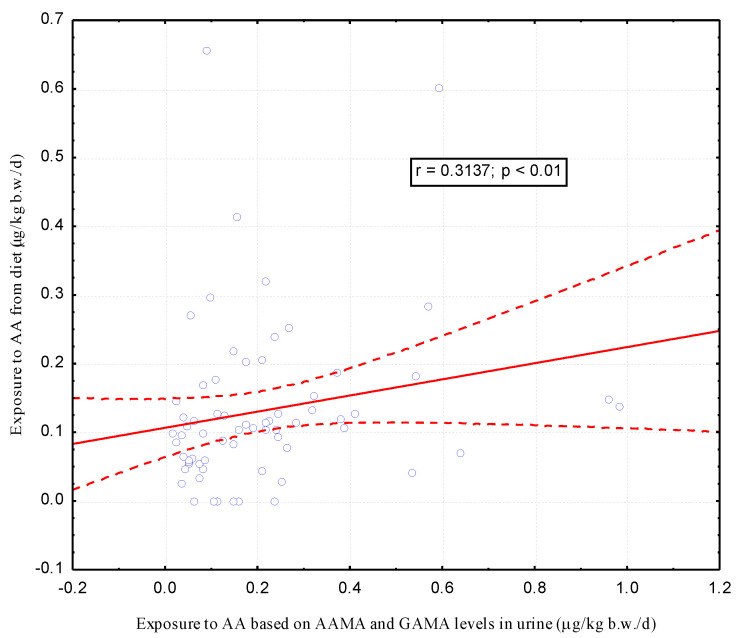
Correlation between acrylamide exposure estimated based on a 24-h dietary recall and AAMA and GAMA levels in urine after childbirth in a group of non-smoking women (n = 67).

**Figure 3:**
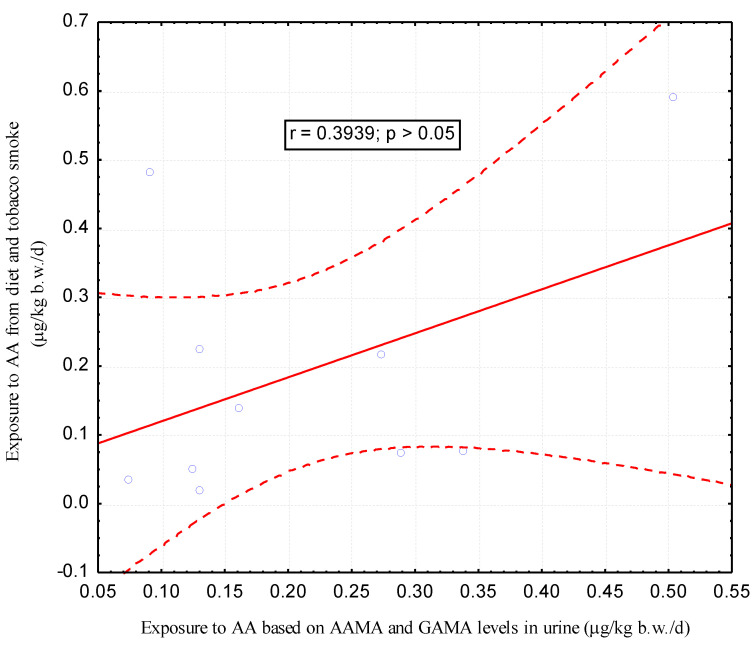
Correlation between exposure to acrylamide estimated based on a 24-h dietary recall after childbirth and passive smoking during pregnancy and AAMA and GAMA levels in urine after childbirth in the group of passive smokers (*n* = 10).

Attention is drawn to the similar level of estimated exposure using two types of approach. However, only in the group of non-smoking women, a significant correlation (r = 0.3137, *p* < 0.01) was found between the results of exposure estimated by two different methods, from 24-h dietary recall and AAMA and GAMA levels in urine (Figure 2 and Figure 3).

Comparison of birth parameters of children of non-smokers and passive smokers during pregnancy (Table 5) shows that there were no significant differences in birth weight and body length between the studied groups. In the case of Apgar scores in the first minute of life, 90% of children of passive smokers and 82.1% of children of non-smoking women scored 10 points, and 10% and 11.9%, respectively, scored 9 points. It should be emphasized that in the subsequent minutes of life (3–5 min) all other newborns of non-smoking women received 10 and 9 Apgar score. 

## 5. Discussion

Despite documented evidence of the harmful effects of smoking on the normal course of pregnancy and the health of the child, some pregnant women smoke and continue smoking after childbirth. In Poland, according to EURO-PERISTAT 2015 [2], the percentage of women actively smoking in the third trimester of pregnancy was 12.3%. Current data presented in the report of the Polish survey on attitudes towards smoking [40] show that Poles still smoke in the presence of non-smokers, pregnant women and children, but this percentage is significantly lower than in 2017. Every fourth smoker (24%) admitted that they smoked in the presence of adult non-smokers, 8% of smokers smoked in the presence of children, 2% in the presence of chronically ill people, and 1% of smokers confirmed smoking in the presence of pregnant women. In our preliminary study, about 13% of women (*n* = 10) admitted that they were exposed to tobacco smoke during pregnancy. One person was exposed sporadically, with an average of 5 cigarettes per month. Three more persons, which accounted for about 30% of all passive smokers, were exposed to daily tobacco smoke from 10 to 20 cigarettes smoked in their presence. The other women were passively smoking 2 to 6 cigarettes every day. 

Tobacco smoke is a complex mixture of more than 5000 chemical compounds of which at least 150 have toxic effects and are collectively referred to as “tobacco smoke toxicants” [41]. Some have been classified by the International Agency for Research on Cancer as carcinogenic (Group 1), probably carcinogenic (Group 2A) and possibly carcinogenic (Group 2B) to humans. Acrylamide is a compound “probably carcinogenic to humans” [21], and its presence in tobacco smoke has been explicitly confirmed [10,11,15]. To estimate pregnant women’s exposure to acrylamide from cigarettes smoked in their presence, we used the results of our own research [15] on the content of this compound in tobacco smoke condensate from the 5 most frequently purchased cigarette brands in Poland. The estimated exposure of pregnant women who were exposed to ≥10 cigarettes per day smoked in their presence was about fivefold (µg/kg b.w./day) significantly (*p* < 0.05) higher than that of women passively exposed to less than 10 cigarettes per day (0.14 µg/kg b.w./day vs. 0.03 µg/kg b.w./day). At the same time, it was similar to the exposure to acrylamide of women actively smoking in Poland estimated in our previous study [15] (0.10 μg/kg b.w./day). 

The main sources of acrylamide exposure to humans are food and tobacco smoke. The hospital diet consumed by the women participating in the study consisted mainly of milk and milk products; cooked and stewed meat and processed meat; cooked potatoes and vegetables; rice and cereal products in the form of bread, bread rolls, and cereal flakes. Additionally, some women also consumed various snacks such as cookies and biscuits and sweets such as chocolate and chocolate products. The beverages were mainly water, tea, fruits juices and chicory coffee. The estimated dietary acrylamide intake in both groups of women varied in a wide but very similar range in non-smokers and passive smokers, 0.00-51.30 µg/person/day and 0.00-49.63 µg/person/day, respectively. The average estimated exposure to dietary acrylamide of passive smokers during pregnancy was 0.06 μg/kg b.w./day and did not differ significantly from that of non-smoking women (0.11 μg/kg b.w./day). The average estimated intake of acrylamide from food, in our study, in the group of passive smokers was about a sevenfold lower and in the case of non-smokers about fourfold lower compared to that estimated, using the Food Frequency Questionnaire (FFQ), by Duarte-Salles et al. [23] in pregnant women in Norway, (0.41 μg/kg b.w./day) and in France in the study of Kadawathagedar M. et al. (0.38 μg/kg b.w./day) [25]. In current research we also found a similar difference to the probabilistic estimation of acrylamide intake in the group of adult women in Poland (0.32 μg/kg b.w./day) in our previous study [42]. It should be emphasized that the women participating in our study stayed in the maternity unit within a few days after the childbirth; they consumed a typical hospital diet, and most of them consumed small amounts of food. Additionally, it should be noted that the hospital diet did not contain the products that provide the highest amounts of acrylamide, such as fried and baked potato products, coffee and coffee substitutes or crispy bread and toast. 

When estimating the total (from diet and tobacco smoke) exposure to acrylamide of passive smokers during pregnancy, it is worth noting that the estimated intake from tobacco smoke increased the intake from diet by more than 50%. However, there was no significant difference in the average estimated exposure to acrylamide from the diet and from tobacco smoke of passive smokers (0.11 μg/kg b.w./day, range: 0.02–0.59 μg/kg b.w./day) and in the exposure to acrylamide assessed exclusively from the diet of non-smoking women (0.11 μg/kg b.w./day, range: 0.00–0.66 μg/kg b.w./day). 

The main metabolites of acrylamide and glycidamide in urine are N-Acetyl-S-(2-carbamoylethyl)-L-cysteine (AAMA) and N-Acetyl-S-(2-carbamoyl-2-hydroxyethyl)-L-cysteine (GAMA), respectively. In our study we wanted to preliminarily check whether the content of acrylamide metabolites (AAMA and GAMA) in women’s urine after childbirth can be a good indicator of acrylamide uptake due to passive smoking during pregnancy. Numerous studies [14,30,31] have shown that active smokers have a significantly higher acrylamide metabolite content than non-smokers, but none of the studies so far presented whether AAMA and GAMA are good markers for assessing exposure to acrylamide from passive smoking in pregnancy. We assumed that the intake of acrylamide from the hospital diet in both groups of women, non-smokers and passive smokers, would be similar. Therefore, differences in AAMA and GAMA content in urine will only be due to exposure to tobacco smoke during pregnancy. In all urine samples after the childbirth of the examined women (*n* = 77), we determined the AAMA and GAMA content by LC-MS/MS method as described earlier (37). In the whole group of women participating in our study, a large variability in the levels of AAMA and GAMA in urine was observed. AAMA content ranged from 2.3 μg/L (7.8 μg/g creatinine) to 114 μg/L (138.2 μg/g creatinine), and GAMA content varied from 1.3 μg/L (4.9 μg/g creatinine) to 50.7 μg/L (26.7 μg/g creatinine). This may be associated with different dynamics of acrylamide conversion to glycidamide and further to mercapturic derivatives, at a similar level of dietary intake. Our results seem to indicate the possibility of an individually determined response to the exposure to acrylamide present in food or the influence of the other sources of exposure to acrylamide, e.g., second-hand smoking. We found greater difference in the minimum and maximum content of the urinary metabolites in the group of non-smokers compared to passive smokers. The median AAMA and GAMA content in urine of pregnant passive smokers after childbirth (*n* = 10) reached values of 21.6 µg/L (25.2 µg/g creatinine) and 9.4 µg/L (10.3 µg/g creatinine), respectively, and was slightly higher than that of non-smokers (*n* = 67), which was 19.5 µg/L (30.7 µg/g creatinine) and 7.2 µg/L (11.4 µg/g creatinine), respectively. However, median of AAMA and GAMA in urine second-hand smoking women did not differ significantly from the content of these metabolites in urine of non-smoking women. Similarly, we found no significant differences in AAMA and GAMA in urine between pregnant women who were exposed to tobacco smoke from more than 10 and less than 10 cigarettes a day in their presence. Similarly, Heudorf et al. [33] did not observe the influence of passive smoking on the level of acrylamide metabolites (AAMA and GAMA) in urine in a group of 110 children aged 5-6 years in Germany. The study compared children in the presence of whom at least one member of the family smoked, or family members smoked at home, to children who were not exposed to tobacco smoke. No significantly higher concentrations of AAMA and GAMA metabolites in urine were found in children exposed to tobacco smoke at home compared to children not exposed to tobacco smoke. Moreover, Brisson et al. [34] did not find any significant differences in the median of urinary levels of AAMA and GAMA in group of Canadian teenagers passively smoking at home compared to teenagers not exposed to tobacco smoke at home. Opposite results were obtained by Ji et al. [35] in a small group of children aged 10-13 years in Korea. The authors found significantly (*p* < 0.05) higher AAMA and GAMA levels in urine of eight children whose parents smoked cigarettes compared to 14 children whose parents did not smoke (98.95 ng/mg creatinine vs. 70.65 ng/mg creatinine). Recently Choi S.Y. et al. [32] stated that the urinary concentrations of AAMA, determined in a nationally representative sample (*n* = 1025) of children and adolescents (age range 3–18 years) in South Korea, increased significantly with passive smoking exposure. They found that passive smoking for > 30 min led to adjusted proportional increases in AAMA of 1.77 times in the non-smoking group aged 3–6 years and a 1.52-fold increase in AAMA in the non-smoking group aged 7–18 years. The lack of differences in AAMA and GAMA content in urine between non-smokers and passive smokers found in our study may be due to several reasons, the first of which seems to be a relatively small group (*n* = 10) of passive smokers. However, it should be emphasized that in our previous study (37), we found significantly (*p* < 0.01) higher levels of AAMA and GAMA in the urine post-partum in a small group of 5 women actively smoking during pregnancy compared to non-smokers. Moreover, in the previously cited study by Ji et al. (35), a significant (*p* < 0.05) difference was found in the levels of AAMA and GAMA, despite a small research group (8 passive smokers vs. 14 non-smokers). A small group of passive smokers during pregnancy is probably due to the overall decline in smoking in Europe and the related difficulties in recruiting a group for research. It is worth emphasizing that in Poland in the eighties of the last century, 42% of Poles smoked tobacco products, but there was a significant reduction in smoking in Poland, to 24% in 2017 [1]. Women mostly stop smoking during pregnancy, and smokers reduced smoking in the presence of pregnant women [40]. The small size of the group of passive smokers was probably also the reason for the lack of significant correlation between the exposure to acrylamide estimated in our studies based on 24-h dietary recall and passive smoking and the exposure estimated based on the level of metabolites in urine in this group of women. Attention is drawn to the very similar values of calculated acrylamide intake based on food consumption data (and content in tobacco smoke in passive smokers) and based on the level of AAMA and GAMA metabolites in urine in the group of passive smokers and non-smokers, respectively, 0.11 μg/kg b.w./day vs 0.11 μg/kg b.w./day and 0.15 μg/kg b.w./day vs 0.16 μg/kg b.w./day. It should be noted that, like Duarte-Salles et al. [23], in the current study, we found a significant (r = 0.3137, *p* < 0.01) positive correlation between exposure estimated from dietary intake of acrylamide (μg/kg b.w./day) and exposure to acrylamide (μg/kg b.w./day) estimated from AAMA and GAMA levels in urine of non-smokers.

Another probable reason, apart from small size of the study group, for the lack of differences in AAMA and GAMA content in urine between non-smokers and passive smokers is the fact that the women tested were not exposed to tobacco smoke for 2 to 5 days while they were in hospital. Therefore, the time elapsed since exposure to tobacco smoke was sufficient for metabolised and eliminated of acrylamide from tobacco smoke from the body. At the same time, this compound was constantly taken from the hospital diet, resulting in similar average levels of AAMA and GAMA in urine for non-smokers and passive smokers during pregnancy. This seems to be confirmed by studies by Bjellass et al. [31], who showed that a one-day of fasting resulted in a rapid, approximately 50% reduction in the total level of acrylamide metabolites in urine. On the other hand, Boettcher et al. [28] showed that although in humans about half of the acrylamide taken is excreted in urine in the form of the AAMA and GAMA metabolites within 24 h, even after 46 h of fasting, the presence of these compounds in urine was still observed. It should be also emphasized that in our previous study [37], we found a significantly (*p* < 0.05) higher AAMA and GAMA content in the urine of women who smoked actively during pregnancy, compared to non-smoking women in pregnancy. Despite the fact that smoking women were also kept in controlled conditions (hospital), and they did not smoke actively, the level of acrylamide metabolites in their urine was several times higher than in those women who did not smoke. This requires further research. 

The key reason for the lack of differences in AAMA and GAMA content in urine between non-smokers and passive smokers during pregnancy, which needs to be confirmed, seems to be difference in the absorption of tobacco smoke in active and passive smoking. An active smoker inhales the main stream of smoke while a passive smoker is exposed to toxic substances contained in the so-called side stream smoke. In a lateral stream of smoke, the concentration of many compounds harmful to health is significantly higher compared to the main stream. It was found that in the side stream the content of nitric oxide is 5 times higher, ammonia and volatile N—nitrosamines 100 times higher, and formaldehyde about 50 times higher than in the main stream [8]. However, acrylamide is mainly present in the particulate phase of tobacco smoke, and its content in the vapor phase is very low [10]. For this reason, the so-called raw condensate is taken from the main smoke stream to determine acrylamide in tobacco smoke. It appears that mainly active smokers are exposed to the acrylamide contained in tobacco smoke, as they inhale the main stream of tobacco smoke. This is perhaps the main reason for the significantly higher AAMA and GAMA content in urine of smokers compared to non-smokers and passive smokers found in previous studies [14,30,31,37]. The answer to the question whether passive smoking may be a source of exposure to acrylamide present in tobacco smoke requires further investigation. First of all, the assessment of the level of acrylamide metabolites in urine of current passive smokers.

Two studies published so far [23,25] have shown a positive correlation between the estimated, using the FFQ questionnaire, intake of acrylamide from food during pregnancy and low birth weight and body length and an increased risk of SGA. Furthermore, Pedersen et al. [24] have shown that the exposure during pregnancy to acrylamide, estimated on the basis of the level of biomarkers, which are adducts with haemoglobin (AA-Hg), is positively correlated with intake of food “rich in this compound”. The authors have shown an inverse correlation between the exposure to acrylamide from the diet during pregnancy and the birth weight of the child and the head circumference. The mechanism of the influence of acrylamide from the diet during pregnancy on the developmental parameters of newborns is not yet sufficiently explained. Acrylamide may penetrate the placental barrier [22] and pose a risk to the developing foetus. Animal studies have shown the effect of acrylamide taken during pregnancy on the reproductive capacity and developmental parameters of the offspring [43]. It should be noted that glycidamide, which is the main and most reactive metabolite of acrylamide, creates a series of DNA adducts, which are formed by the Michael nucleophilic addition reaction [44]. For this reason, glycidamide is considered to play a fundamental role in the carcinogenic effect of acrylamide [45]. Duarte-Salles et al. [23] suggest that glycidamide can form adducts from the foetus DNA, resulting in a disorderly development. However, this hypothesis should be confirmed. 

Passive smoking during pregnancy may be an important risk factor for the child’s normal development and diseases at a later age [7]. However, in our preliminary study we found no significant differences in the length of pregnancy between non-smoking women and passive smokers. Birth parameters of children of non-smoking and passive smokers such as body length and weight also did not differ significantly between the studied groups. The vast majority of newborns (90% in the group of passive smokers and over 80% in the group of non-smokers) scored 10 Apgar points in the first minute of life. All other children in the following minutes (3–5 min) reached a value of 10 or 9. It seems that the lack of differences in child developmental parameters between women who did not smoke and those exposed to tobacco smoke during pregnancy was probably due to a small number of passive smokers. 

## 6. Conclusions

Exposure of pregnant women to acrylamide from passive smoking is still not well documented and requires further research. Our pilot study did not confirm that post-partum AAMA and GAMA concentrations in urine are good markers of exposure to acrylamide from passive smoking during pregnancy. It is probably due to the different ways of acrylamide absorption from tobacco smoke by active and passive smokers. The use of AAMA and GAMA markers of acrylamide in urine of pregnant women currently passively exposed to tobacco smoke is necessary. 

## Figures and Tables

**Table 1:** Characteristics of subjects.

Parameters	Passive Smokers (*n* = 10)	Non-Smokers (*n*= 67)	*p*
Average (range)			
Age (years)	28 (24–35)	31 (21–40)	<0.05
Body weight (kg)	78 (62–95)	72 (51–111)	n.s.
Creatinine content in urine (mg/L)	947 (198–1785)	773 (77–2893)	n.s.
Pregnancy length (weeks)	40 (38–41)	39 (33–41)	n.s.
Number of persons (%)			
Type of labour:			
Natural	5 (50)	44 (65.7)	n.s.
Caesarean section	5 (50)	23 (34.3)	
Labour order:			
First	6 (60)	34 (50.7)	n.s.
Subsequent	4 (40)	33 (49.3)	
Median (min–max)			
Acrylamide intake from the postpartum diet:			
μg/person/day	4.52 (0.00–51.30)	7.33 (0.00–49.63)	n.s
μg/kg b.w./day	0.06 (0.00–0.59)	0.11 (0.00–0.66)	n.s.

n.s.—not significant.

**Table 2:** Estimated exposure of pregnant women to acrylamide from tobacco smoke, depending on the declared daily number of cigarettes smoked in their presence.

<10 Cigarettes (*n* = 7)			≥10 Cigarettes (*n* = 3)			*p*
Exposure to AA		Number of Cigarettes (pcs)	Exposure to AA		Number of Cigarettes (pcs)	
μg/Person/Day	μg/kg b.w./Day		μg/Person/Day	μg/kg b.w./Day		
0.12	0.001	0.2	6.8	0.10	10	
1.36	0.02	2	13.6	0.14	20	
1.70	0.02	2.5	13.6	0.16	20	
1.70	0.03	2.5	-	-	-	
2.72	0.03	4	-	-	-	
3.40	0.05	5	-	-	-	
4.08	0.05	6	-	-	-	
Me = 1.70	Me = 0.03	x = 3.2	Me = 13.6	Me = 0.14	x = 16.7	<0.05

The average content of acrylamide in a cigarette was assumed to be 679.3 ng/cigarette [15]. *n*—number of pregnant women Me—median, x—average.

**Table 3:** Comparison of AAMA and GAMA levels in urine post-partum of non-smoking and passive smoking women during pregnancy.

	Non-Smokers (*n* = 67)		Passive Smokers						p ^*1^	p ^*2^
			In Total (*n* = 10)		<10 Cigarettes (*n* = 7)		≥10 Cigarettes (*n* = 3)			
	Median	Min–Max	Median	Min–Max	Median	Min–Max	Median	Min–Max		
On volume basis [μg/L]										
AAMA	19.5	2.3–114	21.6	9.0–73.9	22.3	9.0–73.9	20.9	10.9–42.1	n.s.	n.s.
GAMA	7.2	1.3–50.7	9.4	3.5–30.9	10.1	5.0–30.9	8.8	3.5–14.1	n.s.	n.s.
AAMA + GAMA	26.9	3.7–164.7	31.0	14.0–104.8	32.4	14.0–104.8	29.7	14.4–56.2	n.s.	n.s.
GAMA − : AAMA	0.38	0.13 – 1.01	0.41	0.26–0.74	0.42	0.26–0.74	0.33	0.32–0.42	n.s.	n.s.
On creatinine basis [μg/g]										
AAMA	30.7	7.8–138.2	25.2	8.6–122.8	25.0	8.6–122.8	25.4	17.5–55.1	n.s.	n.s.
GAMA	11.4	4.9–26.7	10.3	6.4–51.3	10.6	6.4–51.3	8.54	7.3–17.6	n.s.	n.s.
AAMA + GAMA	43.5	15.7–162.0	35.4	15.1–174.1	37.0	15.1–174.1	33.8	24.8–72.7	n.s.	n.s.

^*1^ Comparison of results for non-smokers and passive smokers in total; ^*2^ comparison of passive smokers’ results, depending on the number of cigarettes smoked daily in their presence. n. s.—not significant.

**Table 4:** Comparison of acrylamide exposure to acrylamide of non-smoking and passive smoking women during pregnancy, estimated on the basis of a 24-h dietary recall and on the AAMA and GAMA levels in urine post-partum.

Exposure to Acrylamide Estimated on the Basis of:	[μg/kg b.w./Day]		*p*
	Median (Min-Max)		
	Passive Smokers (*n* = 10)	Non-Smokers (*n* = 67)	
Dietary recall and content in tobacco smoke *	0.11 (0.02–0.59)	0.11 (0.00–0.66)	n.s.
AAMA and GAMA levels in urine	0.15 (0.07–0.50)	0.16 (0.02–0.98)	n.s.

* Exposure to tobacco smoke only applies to women who are passively exposed to tobacco smoke during pregnancy. n. s.—not significant

**Table 5:** Comparison of birth parameters of newborns of non-smoking and passive smoking mothers during pregnancy.

Parameters	Average (Range) or Number of Persons (%)		
	Non-Smokers (*n* = 67)	Passive Smokers (*n* = 10)	*p*
Birth weight (g)	3433 (1860–4750)	3552 (3000–4290)	n.s.
Birth body length (cm)	55 (48–60)	55 (48–58)	n.s.
Apgar score in the first minute of life ^*^:			
10	55 (82.1)	9 (90)	
9	8 (11.9)	1 (10)	
≤8	4 (6.0)	-	

^*^ All children reached 9 or 10 Apgar scores in the 3–5 min.
